# 

**Published:** 1897-06

**Authors:** 


### Southern Medical Record.

A Monthly Journal of Medicine and Surgery.

VOL. XXVII. ATLANTA, GA., JUNE, 1897. No. 6

BERNARD WOLFF, M. D., EDITOR.

ASSOCIATE EDITORS:

A. W. GRIGGS, M. D. WILLIS F. WESTMORELAND, M. D.
J. McFADDEN GASTON, M. D.

E. Y. CLARKE, GENERAL MANAGER. B. M. ZETTLER, BUSINESS MANAGER.

TERMS: $1.00 Per Annum; Single Copy, 10 Cents. Contents on Page 7.

Entered at the Post-Office at Atlanta, Ga., as Second-Class Matter.

The Foote & Davies Co., Atlanta, Ga.

